# Metabolic Differences between Dogs of Different Body Sizes

**DOI:** 10.1155/2017/4535710

**Published:** 2017-10-26

**Authors:** Rondo P. Middleton, Sebastien Lacroix, Marie-Pier Scott-Boyer, Nikola Dordevic, Adam D. Kennedy, Amanda R. Slusky, Jerome Carayol, Christina Petzinger-Germain, Alison Beloshapka, Jim Kaput

**Affiliations:** ^1^Nestlé Purina Research, St. Louis, MO, USA; ^2^The Microsoft Research, University of Trento Centre for Computational and Systems Biology (COSBI), Rovereto, Italy; ^3^Metabolon, Inc., Morrisville, NC, USA; ^4^North Carolina State University, College of Veterinary Medicine, Raleigh, NC, USA; ^5^Nestlé Institute of Health Sciences, Lausanne, Switzerland

## Abstract

**Introduction:**

The domesticated dog,* Canis lupus familiaris*, has been selectively bred to produce extreme diversity in phenotype and genotype. Dogs have an immense diversity in weight and height. Specific differences in metabolism have not been characterized in small dogs as compared to larger dogs.

**Objectives:**

This study aims to identify metabolic, clinical, and microbiota differences between small and larger dogs.

**Methods:**

Gas chromatography/mass spectrometry, liquid chromatography/tandem mass spectrometry, clinical chemistry analysis, dual-energy X-ray absorptiometry, and 16S pyrosequencing were used to characterize blood metabolic, clinical, and fecal microbiome systems, respectively. Eighty-three canines from seven different breeds, fed the same kibble diet for 5 weeks, were used in the study.

**Results:**

449 metabolites, 16 clinical parameters, and 6 bacteria (at the genus level) were significantly different between small and larger dogs. Hierarchical clustering of the metabolites yielded 8 modules associated with small dog size.

**Conclusion:**

Small dogs had a lower antioxidant status and differences in circulating amino acids. Some of the amino acid differences could be attributed to differences in microflora. Additionally, analysis of small dog metabolites and clinical parameters reflected a network which strongly associates with kidney function.

## 1. Introduction

Significant genetic and metabolic variation occurs within the* Canis lupus *species that spans from the wolf through all domesticated canines. Artificial selection for phenotypic traits generated profound genetic differences within* Canis lupus familiaris*, the domesticated dog that is widespread in all human cultures. Dogs can vary in size between roughly 2 and 90 Kg. Segmenting dogs by size reveals some inherent characteristics. Small dogs are more likely to suffer from integumentary, cardiovascular, and dental diseases and have a higher incidence of endocrine-related deaths compared to larger dogs [1, 2]. Small dogs have a lower basal metabolic rate and a higher mass-specific metabolic rate than larger dogs [3]. Interestingly, small dogs have a longer lifespan than larger dogs [4], which differs from what is observed in other mammalian species.

We conducted a diet- and environment-controlled study in canines to understand metabolic, clinical, and microbiota differences between small and larger dogs. Multiple differences were found, specifically in blood concentrations of antioxidants and amino acids, as well as in microbiota composition. To better understand these differences, we identified modules of highly cooccurring metabolites and further analyzed correlations between metabolic and clinical data.

## 2. Materials and Methods

### 2.1. Cohort Study Design

Eighty-three (83) canines from seven different breeds were all fed the same dry extruded kibble diet for 5 weeks. Small dogs (34 individuals; Beagle, Small Fox Terrier, and Miniature Schnauzer) had a mean weight of 9.3 kg (range of 6.1–15.6 kg). Larger dogs (49 individuals; Labrador Retriever, English Setter, Siberian Husky, and Rottweiler) had a mean weight of 31.5 kg (range of 18.4–54.4 kg). Small dogs had a mean age of 6.8 years (range of 2.4–13.3 years) while larger dogs had a mean age of 6.2 years (range of 2.2–9.8 years). All dogs except, Rottweilers, were housed in the same location. Samples were handled and processed in the same manner to avoid technical variability or bias. Plasma-EDTA and serum samples were taken after overnight fasting during the fifth week of feeding. Fecal samples were collected during the fifth week of feeding. Plasma, serum, and fecal samples not immediately analyzed were frozen at −80°C.

### 2.2. Canine Plasma Metabolites

Metabolite profiling was performed as described previously [5]. In summary, metabolites from each blood sample were extracted and analyzed by GC/MS (Thermo Fischer, DSQ mass spectrometer) and LC/MS (Thermo Fischer, LTQ mass spectrometers). We carried out chromatographic separation followed by full-scan MS to record and quantify all detectable ions in the samples [6]. All metabolites with known chemical structure were identified by matching the ions' chromatographic retention index and MS fragmentation signatures with reference library entries created from authentic standard metabolites [7]. The reported masses within the supplemental table represent the respective masses of the ion features utilized to identify each molecule. These ion features could be the mass of the parent molecule, the mass of an ion feature/fragment of the molecule, or an adduct (e.g., sodium adduct) of the parental molecule or an adduct of an ion fragment. Additional library entries were added for ions that were not covered by the standards based on their unique ion signatures (chromatographic and mass spectral) so these ions could then be routinely detected and quantified. For quality control and run-day performance analysis, labeled internal standards were spiked into all samples at different stages of the data acquisition process. The median relative standard deviations were 7% and 12% for the internal standards and endogenous biochemicals, 589 detected in total, respectively.

We identified 589 metabolites in plasma prior to removal of some for low percentage presence across all breeds. Of these, 401 biochemicals matched a named structure in the reference library (named). The remaining 188 biochemicals represented distinct chemical entities that represent a single molecule of discrete molecular formula and structure but that do not currently match a named chemical in the reference library (unnamed).

### 2.3. Canine Clinical Parameters

Most clinical parameters were analyzed in all dogs. Some anthropometric analyses using dual-energy X-ray absorptiometry (DEXA) and thyroid hormones triiodothyronine (T3) and thyroxine (T4) were evaluated only in 34 small dogs and 35 larger dogs (not assessed in Rottweilers). Serum creatinine, creatine kinase, potassium, total bilirubin, total antioxidant status (TAS), aspartate transaminase (AST), gamma-glutamyltransferase (GGT), T3, and T4 were measured using the Cobas c311 or e411 clinical chemistry analyzer, according to manufacturer's directions. Protein digestibility was determined based on amount of protein in food consumed, amount in feces, and corrected for microbial nitrogen [8]. DEXA was performed according to manufacturer's directions.

### 2.4. Gut Microbiota Composition

The sequences for 16S amplicon PCR forward and reverse primers for the variable regions V4 to V6 (V456) were 5′AGGCCAGCAGCCGCGGTAA and 5′GCCRRCACGAGCTGACGAC, respectively. The pyrosequencing was performed using Roche 454 GS-FLX Pyrosequencer. 489,290 sequences were generated for 98 samples (83 samples were used in this analysis). Data quality control and sequence trimming were performed using QIIME's python script “split libraries” [9] with default settings except the following parameters: (i) no barcode mismatches were allowed, (ii) maximum sequence length was set to 520 bp, and (iii) a sliding window of 50 nucleotides was used with average quality score ≥ 25. Pyrosequencing error was removed using flowgram clustering. The chimeric sequences were detected and removed using UCHIME [10]. A total of 265,401 high quality sequences were obtained with the average of 3,198 sequences per sample for the 83 samples used in this analysis. The cleaned sequences were then clustered into operational taxonomic units (OTU) using a closed reference-based OTU picking method with similarity threshold of 97% [10, 11], where the reference data file was obtained from the greengenes website (http://greengenes.lbl.gov, August 2013 release) [11]. A consensus taxonomic assignment for each OTU was performed using the ribosomal database project naïve Bayesian classifier [12] at a minimum confidence interval of 80%. Welch's *t*-test was used to calculate *P* values.

### 2.5. Imputation of Missing Value and Outlier Detection

Parameters (metabolites or clinical) missing more than 20% values across all breeds were discarded while missing values were imputed in cases where less than 20% values were absent. Imputed metabolites were randomly determined from a uniform distribution between 0 and the lowest measured value. Imputed clinical parameters were replaced by the breed-specific median value of corresponding clinical parameter.

Outlier detection was performed using robust principal component analysis (PCA) as described previously [13] and implemented in the* rrcov* package for R [14]. PCA was performed separately for metabolites and clinical variables. One small dog (Small Fox Terrier) was identified as an outlier and removed from subsequent metabolomics analyses. After preprocessing, 449 metabolites (of which 131 were unnamed chemicals) were included in the final metabolomics dataset. No clinical outliers were detected.

### 2.6. Evaluation of Variable Importance

The ability of metabolomics and clinical variables to distinguish between body sizes was evaluated using random forest (RF) with the* rfPermute* package for R [15]. This method not only is an appropriate approach for variable selection [16] but also provides a measure of variable importance. RF model is initially created to calculate variable importance and the response variable is then permuted 1000 times to estimate *P* values for RF importance.

Mann–Whitney *U* tests were also used to compare differences in distribution of measured variables between body size groups in addition to RF. Biomarker studies show that two types of methods may provide complementary results [17]. Metabolite ratio between small and larger dogs was determined by dividing the metabolite value by its median value across all samples, then dividing the small dog mean by the larger dog mean.

### 2.7. Detection of Metabolite Modules and Correlation with Clinical Parameters

Weighted gene coexpression network analysis (WGCNA) implemented in the WGCNA R package [18] was used for determining coexpression patterns between genes. We applied such methodology to metabolomics data to identify groups or modules of highly cooccurring metabolites. Modules were identified using unsupervised clustering Dynamic Branch Cut method with an optimal value of 4 for soft threshold and a minimal number of 10 metabolites per module. Note that modules were randomly assigned colors, while unassigned metabolites were grouped in the grey module. A variable representative of all metabolites within a module—equivalent to the first eigenvalue of the PCA—was obtained* (eigengene)* and Spearman rank pairwise correlations between each* eigengene* and clinical parameters were calculated with unadjusted and Bonferroni Hochberg [19] adjusted *P* value significance thresholds of 0.05 and 0.1, respectively.

To assess the ability of the modules to distinguish between body sizes, a hierarchical tree was constructed with the metabolites from larger dogs and metabolites were colored according to the small dog's module memberships. *Z*-scores were then calculated to evaluate how well a module identified with one population of reference (in this case small dogs) was preserved when the reference was modified (i.e., larger dogs) [20]. Greater *Z*-scores show higher preservation and thus lower ability to distinguish between body sizes. All analyses were conducted with R software version 3.0.1 [21].

Associations with kidney function were performed using Pathway Studio Mammalian, ChemEffect and DiseaseFX databases (Elsevier). Kidney function was represented by the following cell process and clinical parameter terms: kidney function, kidney elimination, kidney excretion, renal reabsorption, kidney tubule function, kidney filtration, kidney blood flow, kidney vascularization, renal vasodilation, renal acidification, renin-angiotensin system, kidney development, renal water reabsorption, renal tubular secretion, renal clearance, and glomerular filtration rate.

## 3. Results

### 3.1. Plasma Circulating Metabolites and Clinical Measures Are Different between Dogs of Different Body Size

449 metabolites were used for statistical and computational analysis (see Supplementary Table 1 in the Supplementary Material available online at https://doi.org/10.1155/2017/4535710). Of these, 131 represent unnamed metabolites. Levels of 66 metabolites, of which 14 were unnamed, were significantly different between small and larger dogs (adjusted *P* value < 0.05; Table 1). 69 clinical parameters were measured in this study (Supplementary Table 2) with 16 being significantly different between small and larger dogs (adjusted *P* value < 0.05; Table 2). Many of these parameters were based on weight (overall weight, DEXA parameters, etc.), which were expected to be different since this study compared two groups of dogs based on weight.

### 3.2. Antioxidant Status Is Lower in Small Dogs

Total antioxidant status, bilirubin, glutathione metabolites, and urate (measures of overall antioxidant status) were lower in small dogs compared to larger dogs (Tables 1 and 2). Total antioxidant status is representative of antioxidants present in the blood that inhibit the oxidation reaction assay. Inhibition can be caused by many types of antioxidants and is thus not specific for an individual antioxidant. Bilirubin is associated with heme breakdown but is also a powerful antioxidant [22]. Glutathione metabolites, including multiple gamma-glutamyl amino acids, cysteine-glutathione, and 5-oxoproline, were lower in small dogs. Glutathione (GSH) is a powerful antioxidant present at high concentrations in cells. Urate, another powerful antioxidant, was also lower in small dogs. Urate, initially produced from xanthine by xanthine oxidase in the metabolism of purines, is converted to allantoin by the uricase enzyme in most species. In humans and great apes, however, the uricase enzyme activity is absent and urate concentrations in these species are elevated [23].

### 3.3. Circulating Levels of Amino Acids Are Different between Dogs of Different Body Size

Several amino acids and their metabolites differed between small and larger dogs (Table 1). Circulating levels of the essential amino acids phenylalanine, tyrosine, lysine, and the nonessential amino acids glutamine, hydroxyproline, and prolylhydroxyproline were lower in small dogs compared with larger dogs. Circulating levels of the essential amino acid arginine were higher in small dogs. Protein digestibility (Table 2) was unexpectedly higher in small dogs even though levels of many circulating amino acids were lower. The amino acid metabolites phenol sulfate and p-cresol-sulfate levels were higher in small dogs. These metabolites are formed in the liver by sulfation of bacterial-derived tyrosine metabolites [24].

### 3.4. Other Plasma Metabolites and Clinical Measures Are Different between Dogs of Different Body Size

Other metabolites and clinical measures involved in biological processes differed between small dogs and larger dogs (Tables 1 and 2). While most of these processes were represented by only one metabolite or clinical measure, they have physiological significance since they function across processes. Creatine levels were higher in small dogs as were two of its building blocks, citrulline and arginine (Table 1). Creatine can be endogenously synthesized by the kidneys, pancreas, and liver, or it can be ingested [25]. Most creatine is phosphorylated and stored as phosphocreatine in skeletal muscle, where it is used to replenish ATP concentrations. Creatine is recycled into creatinine, which is then excreted by the kidney. Creatinine levels were lower in small dogs.

Homocitrulline concentrations were lower in larger dogs. Homocitrulline is formed by the carbamylation of lysine residues and results in loss of the biological activity of lysine [26]. Triiodothyronine (T3) was higher in small dogs. T3 is a thyroid hormone that affects basal metabolic rate and increases O_2_ metabolic consumption affecting both fatty acid and carbohydrate metabolism. Pantothenic acid (vitamin B5), an essential component of coenzyme A, was higher in small dogs. Bone mineral density was lower in small dogs consistent with the results of others [27].

### 3.5. The Fecal Microbiome Differed between Dogs of Different Body Size

**Figure 1:**
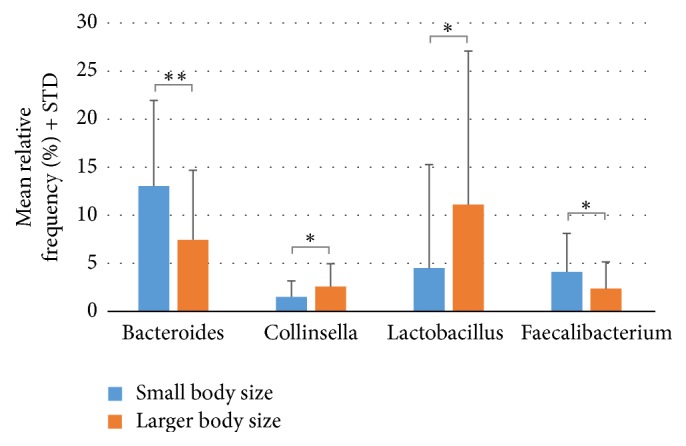
Fecal microbiota differences (genus level) between small dogs and dogs of larger body size. Microbiota differences with *P* value < 0.05 and relative frequencies greater than 1% are shown. ^*∗∗*^*P* < 0.01; ^*∗*^*P* < 0.05.

The fecal microbiome was analyzed to determine if small dogs had differences in bacterial populations compared to larger dogs. Since bacterial populations can change in as little as one day in response to a macronutrient change [28] and all dogs were fed identical diets, fecal samples were taken after 5 weeks, based on the assumption that bacterial populations had normalized. 16S analysis identified 6 bacteria (*P* value < 0.05) at the genus level that differed between small dogs and larger dogs. Of these,* Bacteroides*,* Faecalibacterium* (higher in small dogs), and* Collinsella* and* Lactobacillus* (higher in larger dogs) had the highest mean proportion difference (mean relative frequency > 1%) (Figure 1).

### 3.6. Plasma Metabolites and Clinical Measures Associate with a Kidney Function

**Figure 2:**
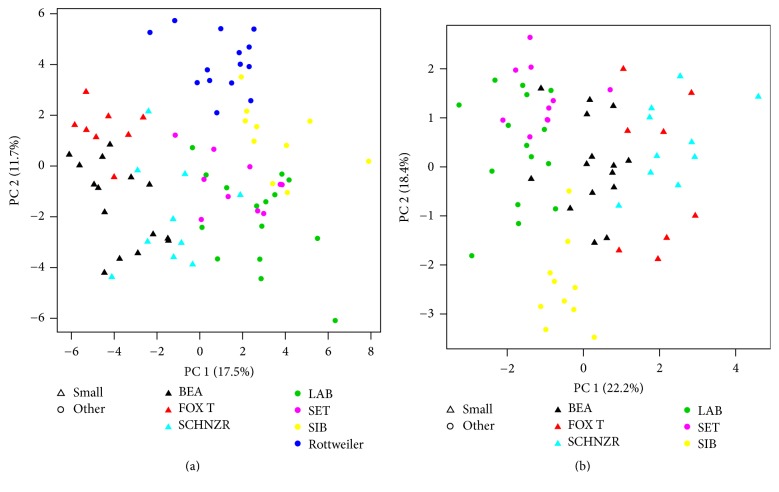
Principle component analysis using selected metabolites (a) and clinical parameters (b) between small dogs and dogs of larger (other) body size. Note that Rottweilers were not considered in the PCA generated with clinical parameters as they are missing DEXA-related parameters along with thyroid hormones T3 and T4. Body size is indicated by triangle (small) or circle (larger, other). LAB, Labrador Retriever; SET, English Setter; BEA, Beagle; FOX T, Small Fox Terrier; SIB, Siberian Husky; SCHNZR, Miniature Schnauzer.

**Figure 3:**
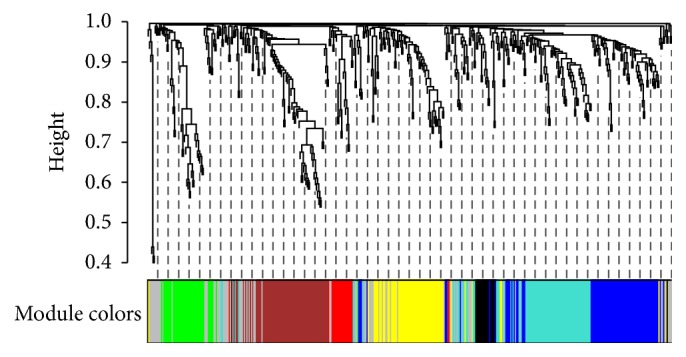
Metabolite dendrogram and module identification. Hierarchical cluster tree of the 449 metabolites, color-coded according to the modules identified using small dogs as the reference.

**Figure 4:**
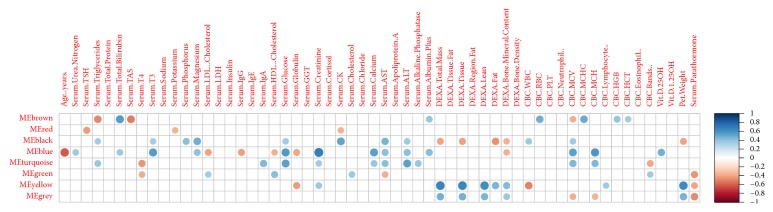
Correlations between metabolite modules and clinical parameters. Significant associations (*P* < 0.05) between metabolite modules and clinical parameters using small dogs as reference are indicated by filled circles. Strength of Spearman correlations detailed by the scale on the right.

Visualization with PCA performed using only selected clinical or metabolite parameters simultaneously confirmed clear discrimination between small and larger dogs (Figures 2(a) and 2(b)). In order to understand relationships between metabolites and clinical parameters, metabolite small dog modules were first identified with WGCNA using the 449 metabolites (Figure 3). 8 modules were identified (Table 3). The blue module containing 64 named metabolites (Supplementary Table 3) had the highest ability to distinguish between small dogs and larger dogs (lowest *Z*-score and thus lowest preservation when the reference group was changed). A correlation analysis was then performed between the clinical parameters and metabolomics modules* eigengenes* (Figure 4). Age (negative), creatinine, and bilirubin (both positive) among other clinical parameters showed significant correlations (Figure 4) with blue module metabolites. Metabolites and clinical parameters were analyzed for associations with kidney function. Greater than 30% of the metabolites (Supplementary Table 4) and nearly all blue module associated clinical parameters (Supplementary Table 5) have associations with kidney function.

## 4. Discussion

Although Monod's dictum that anything true of* E. coli* is also true of the elephant describes well the commonality of regulatory and biochemical reactions across the tree of life, subtle and important differences in transcriptional and metabolic processes occur between species and within individuals of the same species. Analysis of plasma metabolites and the microbiome revealed differences based on body size between small and larger dogs. Hierarchical clustering of the final, preprocessed metabolites identified metabolites with similar concentrations (i.e., the blue module) that had the highest ability to distinguish between small and larger dogs. This module contained 64 named metabolites in different metabolic classes (amino acid, nucleotides, carbohydrates, xenobiotics, and cofactors; see Supplementary Table 3).

Small dogs have lower plasma levels of multiple antioxidants, including total antioxidant status, urate, glutathione metabolites, and bilirubin as compared to larger dogs suggesting a higher mass-specific metabolic rate [3] in tissues. Lower levels of antioxidants may be an adaptation to a higher production of free radicals because of the higher basal metabolic rate in small dogs. Bilirubin exerts its most potent antioxidant effects against lipid oxidation, while water-soluble urate and glutathione have much more potent antioxidant effects on protein [29], which when considered together indicated a systemic difference between small and larger dogs.

Urate concentrations were higher in larger dogs compared with small dogs. Urate has both anti- and prooxidant characteristics. Urate utilizes glutathione to reduce brain free radicals, by increasing cysteine uptake via EAAT-1 transporters in neurons of the hippocampus [30]. Urate has specifically demonstrated protective effects against peroxide [31], 1-methyl-4-phenyl-pyridinium [32], the reaction products of peroxynitrite and CO_2_, CO_3_^−^, and NO_2_ [33], as well as 6-hydroxydopamine (6-OHDA) [34]. Appropriate urate levels have protective effects against oxidative diseases such as Alzheimer's Disease [35], Parkinson's disease [36], and Multiple Sclerosis [37].

However, urate can also be prooxidant. A 1 mg/dL increase in serum urate concentrations has been associated with a significant increased incidence of hypertension [38]. Higher urate concentrations have also been shown to predict worsening of renal disease in patients with renal failure [39] and affect glomerular filtration rate in healthy study participants [40]. Proper regulation of urate concentration is thus important to mediate its anti- or prooxidant properties.

Glutathione is present in all cells in high concentrations and acts as a buffer in redox reactions. GSH is the most prevalent antioxidant in the liver, is stored in its reduced state, and protects hepatocytes against oxidative damage. GSH is involved in maintaining thiol disulfide balance, peroxide detoxification, leukotriene biosynthesis, and amino acid transport and has been shown to play a role in multiple cellular processes such as transcription, proliferation, and apoptosis [41]. Cardiac disease is associated with decreased GSH levels in dogs [42]. Compared with healthy dogs, GSH concentrations in clinically ill dogs are significantly decreased in erythrocytes, and GSH depletion correlates with severity of illness and mortality [43]. We showed here that multiple metabolites of GSH metabolism were lower in the plasma of small dogs suggesting utilization for GSH production. However, the true response of the GSH system in small dogs is not known since oxidative states of the GSH were not measured in this study.

We have shown that bilirubin concentrations were lower in small dogs. Bilirubin represents a lipophilic cytoprotectant antioxidant which is complementary to water-soluble antioxidants, such as GSH [29]. The clinical implications of low bilirubin in small dogs are unclear, although lower concentrations suggest increased metabolic consumption and the possibility of ongoing oxidative damage in small dogs.

Small dogs had lower levels of multiple amino acids, including the essential amino acids phenylalanine, tyrosine, and lysine. Arginine was an exception since it was found at higher levels in small dogs. We previously determined that protein digestibility was higher in small dogs suggesting that metabolic differences produced the lower levels of amino acids in small dogs.

Phenylalanine is involved in the synthesis of tyrosine, which is then used as a precursor for the synthesis of adrenaline, noradrenaline, and dopamine [44]. Tyrosine can be fermented in the large intestine, resulting in the microbial formation of* p-*cresol and indoxyl (a phenolic compound), which after hepatic sulfation become* p-*cresyl sulfate and phenol sulfate [24, 45]. Concentrations of both of these compounds were higher in small dogs suggesting that phenylalanine and tyrosine were lower due to fermentation and sulfation, perhaps through increased microbial metabolism. Levels of certain microbial populations affect formation of* p*-cresyl sulfate indicative of differences in the gut microflora between small and larger dogs [45]. 16S analysis revealed higher levels of* Bacteroides* in small dogs.* Bacteroides fragilis* has been shown to produce phenols from tyrosine [45]. Previous studies have shown differences between fecal versus illeal protein digestibility which takes into account microbial contributions to amino acid metabolism [46, 47] consistent with the results presented here.

We observed higher levels of arginine and lower levels of lysine in small dogs. Lysine and arginine share the cationic amino acid transporter system (CAT) mechanism, and concentrations of one competitively inhibit transport of the other which has been observed in mammalian cells [48, 49]. The clinical implications of differences in concentrations of these amino acids cannot be interpreted with the current data, although arginine supplementation is known to be beneficial in wound healing and is essential for maintaining intestinal integrity [50]. Additionally, the lower levels of plasma glutamine in small dogs may be due to absorption into enterocytes, since enterocytes use glutamine as their primary fuel source [51].

Creatine concentrations in plasma were lower in larger dogs. In addition to dietary source, creatine can be synthesized endogenously and is stored as phosphocreatine in muscles as an ATP buffer. Creatine is excreted as creatinine in urine and often serves as a marker of kidney function. Dogs with increased body weight have increased urinary creatinine excretion, suggesting increased creatine turnover, consistent with the higher levels of creatinine in larger dogs noted in our study [52]. However, creatinine may also be associated with increased muscle mass or muscle volume. Sighthounds have more muscle volume than breeds of similar body weight with concomitant increased creatinine concentrations when compared with other breeds [53, 54]. Reduced concentrations of creatine might be associated with increased storage as phosphocreatine, increased usage in skeletal muscle, and consequent increased creatinine excretion.

Plasma homocitrulline was higher in larger dogs. Elevated homocitrulline concentrations have been correlated with the progression of renal disease [55, 56]. Homocitrulline concentration is inversely associated with estimated glomerular filtration rate [55, 57]. Kidney disease has a prevalence of 0.5–1.5% in the canine population [58]. In a retrospective study in Sweden an average of 15.8 cases of kidney disease were diagnosed per 10,000 dog years at risk (DYAR), with a mortality of 9.7 deaths per 10,000 DYAR [59]. Of the breeds exceeding the mean incidence of kidney disease, 23 are considered breeds of larger body size. However, the three breeds with the lowest incidence of mortality due to kidney disease were all small breeds. This is especially interesting because a different survey-based study found that smaller dogs are at greater risk for kidney disease: with every decrease of 10 kg in body weight, a 50% increased risk of kidney disease was found [60].

Many other metabolites in the blue module were associated with kidney function (Supplementary Table 4). Other clinical parameters correlated with metabolites in this module also have associations with kidney function (Supplementary Table 5). Based on these data, this metabolite module and its clinical correlates may contribute to a kidney metabolite network. Many of the differences noted here between small and larger dogs can be attributed to functional differences rather than disease since the animals used in this study did not have clinical kidney conditions. However, no predispositions are known between the small and large breed dog populations, but more research needs to be done here to determine the clinical significance.

## 5. Conclusions

We have described here metabolic differences between small dogs and dogs of larger body sizes. This included the difference in circulating metabolites, clinical parameters, and microbiota from dogs in a diet and environment-controlled study. Small dogs had a lower antioxidant status as measured by multiple metabolites and clinical parameters. Differences were also shown in circulating amino acids, some of which could be tied to variations in specific bacteria of the microbiota. Additionally, analysis of small dog metabolites and clinical parameters reflected a network which strongly associates with kidney function. This analysis represents a unique and initial view of metabolic differences between body sizes within a mammalian species. These differences reflect not only morphometric induced variability, but also metabolic-specific genetic differences associated with the creation of breeds due to artificial selection. More will need to be done in order to identify other unique metabolic characteristics of small dogs including studies representing more breeds belonging to the body size classes of* Canis lupus familiaris*.

## Supplementary Material

Supplemental Table 1 provides the information for all 449 metabolites measured. Supplemental Table 2 provides information for clinical parameters measures. Supplemental Table 3 provides the information for metabolites in the blue module. Supplemental Table 4 provides the association of the blue module metabolites with kidney function. Supplemental Table 5 provides the association of blue module associated-clinical parameters with kidney function.

## Figures and Tables

**Table 1:** Plasma metabolites with adjusted *P* value < 0.05 between small dogs and dogs of larger body size.

Metabolite name	Ratio (small/larger)	Class	Adj. *P*	RI	Mass
Creatine	2.81	Amino acid	7.35*E* − 08	758	132.1
Creatinine	0.78	Amino acid	2.47*E* − 07	730	114.1
5-Oxoproline	0.78	Amino acid	3.28*E* − 07	744	128.2
gamma-Glutamylphenylalanine	0.71	Peptide	3.66*E* − 06	2846	295.1
X-18487	0.48	Unnamed	5.41*E* − 06	1269.6	273.1
Hydroxyproline	0.72	Amino acid	7.97*E* − 05	705	132.1
Phenylalanine	0.84	Amino acid	7.97*E* − 05	2056	166.1
X-14625	0.82	Unnamed	7.97*E* − 05	742	308.1
X-17381	2.94	Unnamed	7.97*E* − 05	4159.8	293.1
p-Cresol sulfate	1.48	Amino acid	1.05*E* − 04	2896	187.1
X-11334	0.47	Unnamed	1.19*E* − 04	982	259.1
Urate	0.72	Nucleotide	1.58*E* − 04	1928	441.2
X-13731	1.92	Unnamed	3.57*E* − 04	1902	235
gamma-Glutamylisoleucine	0.78	Peptide	4.15*E* − 04	2644	261.2
gamma-Glutamylleucine	0.76	Peptide	4.15*E* − 04	2744	261.2
gamma-Glutamylvaline	0.77	Peptide	4.64*E* − 04	2040	247.2
Pseudouridine	0.89	Nucleotide	4.96*E* − 04	1104	243.1
Phenol sulfate	1.74	Amino acid	6.12*E* − 04	2150	173.1
X-12668	1.72	Unnamed	6.76*E* − 04	2318	246.1
C-Glycosyltryptophan	0.79	Amino acid	9.31*E* − 04	1912	367.1
myo-Inositol	0.79	Lipid	0.001	1924.9	217
17-Methylstearate	1.42	Lipid	0.001	5987	297.4
X-14314	0.79	Unnamed	0.001	2302	241.1
Glutamine	0.89	Amino acid	0.002	684	147.2
X-12010	0.72	Unnamed	0.002	1707	203.1
Glycolate (hydroxyacetate)	0.87	Xenobiotics	0.002	1119	177
gamma-Glutamyltyrosine	0.75	Peptide	0.002	2073	311.2
X-12822	0.62	Unnamed	0.004	2786	389.1
Xylonate	0.58	Carbohydrate	0.004	1722	292
Prolylhydroxyproline	0.19	Peptide	0.005	960	229.2
Mannitol	0.33	Carbohydrate	0.005	1839	319.1
Hydroquinone sulfate	1.57	Xenobiotics	0.005	1383	189
Ethanolamine	0.61	Lipid	0.005	1304	174.1
4-Ethylphenyl sulfate	1.50	Xenobiotics	0.006	3570	201.1
Arabonate	0.69	Cofactors and vitamins	0.006	1736	292.1
N6-Carbamoylthreonyladenosine	0.87	Nucleotide	0.006	2656	413
Pantothenate (Vitamin B5)	1.32	Cofactors and vitamins	0.006	2218	220.1
Pyroglutamine	0.74	Amino acid	0.006	764	129.2
gamma-Glutamylmethionine	0.77	Peptide	0.008	1993	279.2
X-16940	3.45	Unnamed	0.010	1694.1	204.9
Citrulline	1.21	Amino acid	0.010	715	176.1
Tyrosine	0.86	Amino acid	0.010	1516	182.1
Gulono-1,4-lactone	0.68	Cofactors and vitamins	0.011	1862	333.1
Methylpalmitate (15 or 2)	1.23	Lipid	0.011	5698	269.4
X-16394	0.79	Unnamed	0.011	1719	229.2
Xylitol	0.75	Carbohydrate	0.014	1677.6	217
Arginine	1.15	Amino acid	0.015	728	173.2
2′-Deoxycytidine	0.84	Nucleotide	0.021	1256	228
2′-O-Methylguanosine	0.59	Nucleotide	0.022	1926	298
Ophthalmate	0.47	Amino acid	0.023	1457	290.1
Homocitrulline	0.77	Amino acid	0.024	832	190.1
5-Methylcytidine	1.13	Nucleotide	0.025	1388	258
N-Formylmethionine	0.89	Amino acid	0.029	1541	176.1
Bilirubin (E,E)	0.50	Cofactors and vitamins	0.031	4625	585.2
X-17299	0.83	Unnamed	0.031	1265.9	229.2
X-18156	0.79	Unnamed	0.031	1392	272.1
Palmitoyl sphingomyelin	0.84	Lipid	0.032	2524	311.3
X-16945	1.73	Unnamed	0.036	3457.9	351
Cysteine-glutathione disulfide	0.81	Amino acid	0.038	821	427.1
4-Vinylphenol sulfate	1.29	Xenobiotics	0.040	3323	199.1
Erythritol	0.87	Xenobiotics	0.040	1517.5	217
Dihomolinolenate (20:3n3 or 3n6)	1.19	Lipid	0.043	5600	305.4
Anthranilate	1.36	Amino acid	0.049	3213	138.1
Lysine	0.75	Amino acid	0.049	1836.7	317.2
Threitol	0.86	Carbohydrate	0.049	1513	217.1
Threonate	0.74	Cofactors and vitamins	0.049	1560.7	292.1

Ratios of median transformed values, Benjamini-Hochberg adjusted *P* values, metabolite biological class, retention index (RI), and mass (Da) are shown. X-##### metabolites represent distinct chemical entities of discrete molecular formula and structure but do not currently match a named biochemical in our reference library.

**Table 2:** Clinical measures with adjusted *P* value < 0.05 between small dogs and dogs of larger body size.

Clinical measure	Mean small	SEM small	Mean larger	SEM larger	Adj. *P*
DEXA tissue (gm)	9135.12	401.87	25392	569.82	1.34*E* − 18
DEXA lean (gm)	6884.76	308.56	17942.89	491.91	1.34*E* − 18
DEXA bone mineral content (gm)	379.58	15.99	1009.06	24.88	1.34*E* − 18
Weight (gm)	9486.15	421.58	31768.72	1302.21	2.2*E* − 16
DEXA fat (gm)	2250.42	145.25	7449.06	427.25	2.91*E* − 16
DEXA total mass (kg)	9.51	0.42	26.40	0.59	1.27*E* − 11
Bone density (gm/cm^2^)	0.65	0.01	0.78	0.01	3.07*E* − 10
Serum creatinine (mg/dL)	0.64	0.02	0.86	0.02	7.80*E* − 07
Serum creatine kinase (IU/L)	263.88	33.51	154.04	21.37	5.04*E* − 05
Serum potassium (mmol/L)	4.31	0.05	4.65	0.05	9.33*E* − 04
Serum total bilirubin (mg/dL)	0.10	0.01	0.13	0.01	0.02
Serum aspartate transaminase (U/L)	35.68	1.99	27.91	1.07	0.02
Serum gamma-glutamyltransferase (g/dL)	6.93	0.43	5.94	0.36	0.02
Serum total antioxidant status (mmol/L)	1.49	0.03	1.60	0.02	0.03
Serum T3 (nmol/L)	1.06	0.04	0.94	0.03	0.04
Protein digestibility (percentage)	88.00	0.38	86.00	0.57	0.01

Mean, standard error of the mean (SEM), and adjusted *P* values are shown. DEXA, dual-energy X-ray absorptiometry; T3, total triiodothyronine.

**Table 3:** Metabolite module preservation and ability to distinguish between small dogs and dogs of larger body size.

Module	Size	*Z*-score
Brown	70	20.61
Green	42	13.97
Yellow	63	9.38
Turquoise	82	7.52
Red	21	5.76
Grey	72	4.22
Black	19	4.11
Blue	80	1.51

*Z*-scores associated with each metabolite module. Greater *Z*-scores depict greater preservation when the reference is changed from small to larger dogs. Thus, modules with greater *Z*-scores have a lower ability to distinguish between groups.

## Abbreviations

AST: Aspartate transaminase
DEXA: Dual-energy X-ray absorptiometry
DYAR: Dog years at risk
GC/MS: Gas chromatography/mass spectrometry
GGT: Gamma-glutamyltransferase
GSH: Glutathione
LC/MS: Liquid chromatography/tandem mass spectrometry
OTU: Operational taxonomic units
PCA: Principal component analysis
RF: Random forest
T3: Triiodothyronine
T4: Thyroxine
TAS: Total antioxidant status
WGCNA: Weighted gene coexpression network analysis.
